# A quantitative analysis of therapeutic cancer vaccines in phase 2 or phase 3 trial

**DOI:** 10.1186/s40425-015-0093-x

**Published:** 2015-11-17

**Authors:** Amabel CL Tan, Anne Goubier, Holbrook E. Kohrt

**Affiliations:** Peter Doherty Institute for Infection and Immunity, Department of Microbiology and Immunology, The University of Melbourne, Melbourne, Australia; PX Biosolutions, Melbourne, Victoria 3205 Australia; DROIA, Meise, 1860 Belgium; Department of Medicine, Division of Oncology, Stanford University, CCSR 1110, 269 Campus Drive, Stanford, California 94305 USA

**Keywords:** Vaccine, Immunotherapeutic, Cancer, Clinical trial

## Abstract

Despite the progress that has been made in other forms of cancer therapy, Provenge® (Sipuleucel-T) is the only FDA-approved vaccine for the treatment of cancer. To understand the current landscape of therapeutic oncology vaccines we performed a quantitative analysis of phase 2 and phase 3 therapeutic cancer vaccine trials. We highlight shifts in trends for the vaccine platforms examined, common adjuvant use, target indications, antibody or treatment combinations between past and recent trials as well as discuss the relationship between these trends and ratio between the number of phase 3: phase 2 for different vaccine platforms. Despite the poor success rate in vaccine approvals, registration of phase 3 trials between 2010 and 2014 were stable indicating continued investment and efforts towards development of immunotherapeutic vaccines.

**Electronic supplementary material:**

The online version of this article (doi:10.1186/s40425-015-0093-x) contains supplementary material, which is available to authorized users.

## Introduction

Significant efforts have been made towards the development of therapies to control or eradicate cancer, a disease that currently kills 8 million people annually (http://globocan.iarc.fr). Between 1996 and 2014, the FDA approved 175 drugs for the treatment of various indications of oncology, 69 of which were approved in the last 5 years (2009–2014) (data obtained from CentreWatch). These therapies include the angiogenesis inhibitor Avastin, the monoclonal anti-HER2/neu receptor antibody, Herceptin and the checkpoint inhibitor anti-PD1. In spite of the significant progress in other forms of cancer therapy and large number of vaccine trials conducted, to this day only one immunotherapeutic cancer vaccine has received FDA approval, the autologous dendritic-cell based immunotherapy Provenge® (Sipuleucel-T) for the treatment of metastatic castrate resistant hormone refractory cancer.

In this review, information on immunotherapeutic cancer vaccine trials was examined to identify trends in the current portfolio of investigational vaccines and highlight the shifts in the focus in vaccine efforts over time. To this aim, data on therapeutic cancer vaccines was obtained from clinicaltrials.gov. registry and medtrack (downloaded on 27th October 2014) using the search terms “vaccine” and “cancer” or “oncology” and entries were consolidated to remove duplicate entries. Analysis was performed on 451 data entries of immunotherapeutic vaccines in phase 2 trials (not including Phase 1/2) and phase 3 trials. We evaluated the conditions targeted, vaccine modalities and adjuvants and combinations concurrently employed. Studies investigating preventative vaccines such as Gardasil, non-cancer vaccines or immunotherapy with BCG were excluded. For a full analysis of all cancer vaccines tested since 2008, inclusive of phase 1–3 trials, see Ref [13].

## Review

### The current landscape of vaccines in phase 2 and 3 clinical trial

**Fig. 1:**
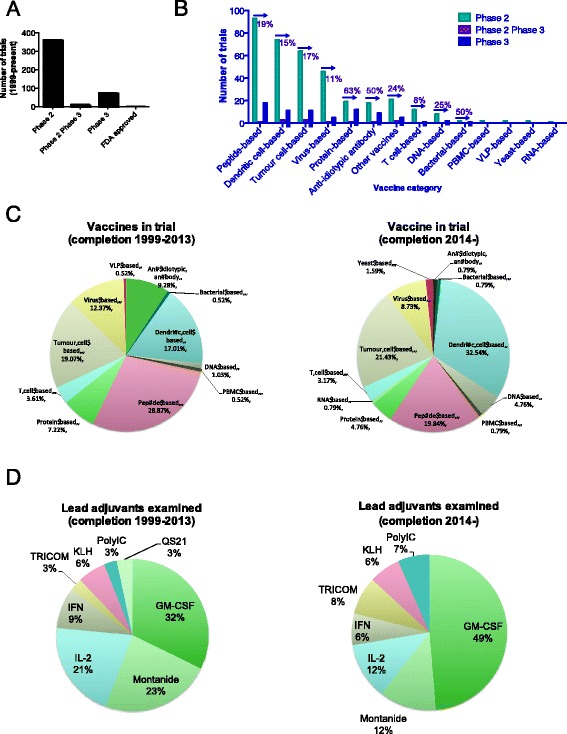
Summary of immunotherapeutic vaccine trials registered since 1999 to 2014. A, Total number of immunotherapeutic anti-cancer phase 2, phase 2 & 3 and phase 3 trials in dataset collected on 27th October 2014. B, Breakdown of the vaccine categories under investigation. Values above or below the arrow indicate the ratio of trials in Phase 3: Phase 2 for the vaccine category (# phase 3 trials/# phase 3 trials *100). C, Comparison of vaccine categories examined in trials completed between 1999–2013 and in 2014 onwards. D, Frequency of the most used adjuvants in vaccine trials completed between 1999–2013 and in 2014 onwards

Of the 451 trials examined, a substantially larger proportion of phase 2 trials were registered compared to phase 3 trials (Fig. 1a). The fewer number of phase 3 trials could be due both to a lack of progression of vaccines from phase 2 to phase 3 (see below for further discussion) as well as the consolidation of activity to multi-centre trials collectively enrolling a larger number of patients. Consequently, we found a higher overall number of patients enrolled in phase 3 cancer vaccine trials (27,141 patients) versus the number enrolled in phase 2 or phase 2/3 trials (20,042 patients), similar to what has recently been reported [13].

### Vaccine modalities under clinical investigation

Based on the information provided in the study records (clinicaltrials.gov), company websites and linked publications the range of vaccine modalities in trial were evaluated and included peptide-based, dendritic cell-based, tumor cell-based, virus-based, protein-based, anti-idiotypic antibody, immunotherapy, T cell-based, DNA-based, bacterial-based, PBMC-based, VLP-based, yeast-based and RNA-based vaccines, in order of prevalence (Table 1). Vaccines that included a cellular or immune stimulatory component that could not be definitely assigned to the other categories (including cytokine activated lymphocytes/cells) have been classified as “other type of vaccines”. Peptide-based, dendritic cell and tumor-based vaccines dominated in all phases hinting towards little novelty in the types of vaccines under trial (Table 1 and Fig. 1b). However, by assigning more granular typing to these vaccine categories we reveal high diversity within each category such as autologous versus allogenic dendritic cells, number of antigens (e.g. single or multi-peptides), use of adjuvant and antigen loading systems (peptide, protein or various virus-based), highlighting the complex and diverse number of features incorporated into the vaccination regime within the larger category (Additional file 1: Table S3).

**Table 1:** Categories of vaccines and immunotherapeutic studies in Phase 2 or Phase 3 trial

Immunotherapeutic and vaccine studies under investigation					
Vaccine category	Phase 2	Phase 2/3	Phase 3	Total^a^	Ratio of Phase 3:Phase 2^b^
Peptide-based	93	1	18	112	1:19
Dendritic cell-based	74	3	11	88	1:15
Tumour cell-based	64	3	11	78	1:17
Virus-based	46	1	5	52	1:11
Protein-based	19	1	12	32	1:63
Anti-idiotypic antibody	18	1	9	28	1:50
Others	21	2	5	28	1:24
T cell-based	12		1	13	1:8
DNA-based	8		2	10	1:25
Bacterial-based	2		1	3	1:50
PBMC-based	2			2	NA
VLP-based	2			2	NA
Yeast-based	2			2	NA
RNA-based	1			1	NA
Grand Total	364	12	75	451	1:21

^a^Sorted by prevalence

^b^ratio of phase 3 trials: phase 2 trials

Closer examination of the 347 studies with primary completion dates (Figure 1c, Additional file 1: Table S1) showed that prior to 2014, the largest proportion of studies examined peptide-based vaccines whilst studies with anticipated completion in 2014 or after are mostly dendritic cells based vaccines, indicating some shift in the focus of efforts (Figure 1c). Given the only FDA approved vaccine Provenge is a dendritic-cell based immunotherapy it will be interesting to see what emerges from the large number of studies investigating the dendritic-cell based platform.

To understand if the prevalence of dendritic-cell based vaccine efforts is due to better progression to phase 3 trials we calculated ratios for phase 3:phase 2 trials as an estimation for the transition rate for each vaccine category (Table 1 and Fig. 1b). Interestingly, this shift in efforts away from peptide-based vaccines towards dendritic cell-based vaccines would not have been predicted based on the transition of these vaccine categories from Phase 2 to Phase 3. Based on ratio values, phase 3 trials were as likely to involve peptide-based vaccines as dendritic-cell based vaccines (Table 1 and Fig. 1b). The same was true when the earlier (pre 2013) and later trials (post 2013 completion) were examined separately (Additional file 1: Table S1). In another example, despite the relatively high ratio of Phase 3: Phase 2 trials for protein-based vaccines we saw a reduction in the contribution of these trial post 2013 indicating that historical progression to phase 3 did not predict for a likely hood of present-day activity of this vaccine type. Similarly, while anti-idiotypic antibody vaccines showed high ratio of phase 3: phase 2 trials overall, we have evidenced a recent drop in the number of anti-idiotypic vaccines being investigated. This is possibly due to the failure of anti-idiotype vaccines mitumprotimut-T [5] and abagovomab [20] to improve overall survival (OS) or time to progression (TTP) in phase 3 trials despite the touted success in earlier studies.

Overall we found that ratio of trials in Phase 3: Phase 2 did not necessarily reflect the shift in efforts within a vaccine category.

Regarding adjuvant selection, GM-CSF and Montanide were the most commonly included followed by IL -2, KLH, TRICOM, QS21 and Poly-ICLC (Table 2 and Fig. 1d). GM-CSF was often also used in combination with Montanide or other adjuvants including QS21, TRICOM, and cytokines such as IL-2 and the protein carrier-based adjuvant Keyhole limpet hemocyanin (KLH). A phase 1 trial examining overlapping NY-ESO-1 long-peptides has revealed that while Montanide was able to elicit high-avidity CD4+ T cell precursors, the addition of Poly-ICLC suppress the induction of Th2 and IL -9 producing Th9 cells. These findings indicating the potential cooperative benefits for combining these commonly examined adjuvants to enhance the Th1 polarisation of vaccine-induced T cells in vaccine studies [23], a strategy that may improve the efficacy of well known adjuvants.

**Table 2:** Most commonly used adjuvants in therapeutic cancer trials

Number of studies including most commonly used adjuvants/immunostimulants				
Adjuvant	Phase 2	Phase 2/3	Phase 3	Total
GM-CSF	92	1	16	109
Montanide	60	1	5	66
IL-2	53		3	56
KLH	16		8	24
TRICOM	10		3	13
QS21	5		1	6
Poly-ICLC	8	0	0	8

Considering the large number of studies examining GM-CSF (109 of the 451 studies) there is no clear information on the benefit of GM-CSF inclusion in the outcome of vaccination with a number of studies finding no benefit to the induction of immune responses or clinical endpoints [8, 13, 17]. A more detailed discussion can be found in the review by Kaufman et al., who report on the inconsistency of results achieved with studies utilizing GM-CSF in immunotherapy for the treatment of melanoma [13]. In this review only four trials included GM-CSF controls when examining its adjuvant activity in vaccination, with two studies observing minimal adjuvant effect and the final two showing diminished activity compared to IFA or BCG [13]. Overall there have been a limited number of studies formally examining the impact of GM-CSF as an active control in a ‘randomized’ trial setting (NCT00324831, NCT00524277, NCT00769704) though at least 30 trials (non-randomised, randomized or treatment design) include GM-CSF alone as a form of treatment indicating that there are efforts to delineate the baseline activity of GM-CSF. This includes 8 ongoing trials (including a phase 3 trial) (Thomson Reuters Cortellis™ database). Interestingly, despite the lack of consistent evidence for activity, especially in phase 3, we did not observed any decrease in the frequency of trials with GM-CSF as an adjuvant, on the opposite (see Fig. 1c).

### Target indications

**Fig. 2:**
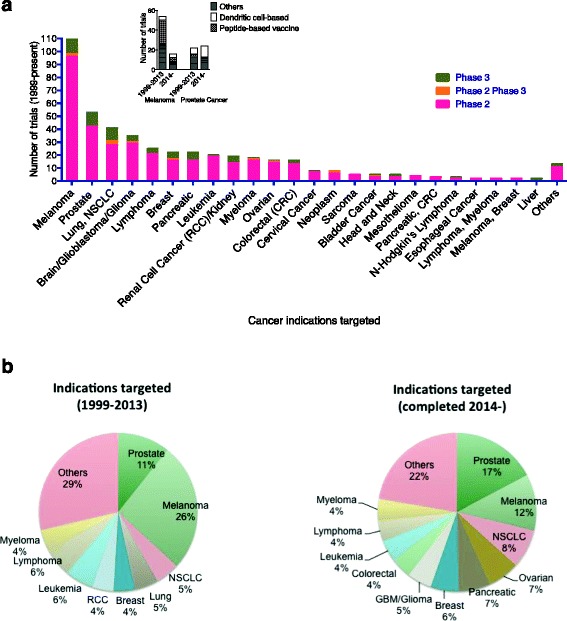
Cancer indications under investigation in Phase II and Phase III trials. **a** Indications are sorted from the most evaluated (left) towards the least (right) using a total of 451 studies. The insert shows the prevalence of studies involving peptide-based or dendritic cell based vaccine in studies completed prior to 2014, or after 2014 (in the 347 studies where completion data was available). **b** Indications targeted in trials prior to 2014, and after 2014. The % value indicates the percentage contribution for the total number of vaccine trials examined

The WHO currently reports that the major cancer killers are lung cancer, liver cancer, stomach cancer, colorectal cancer, breast cancer and oesophageal cancer. Melanoma was the lead indication targeted with immunotherapeutic vaccines followed by prostate cancer, lymphoma, breast cancer, non-small cell lung cancer (NSCLC) and pancreatic cancer and a similar (though not identical) spread between Phase 2 and Phase 3 trials. With the exception of melanoma, the indications targeted closely represent the lead indications implicated in worldwide mortality (Fig. 2a).

A large number of melanoma trials utilized peptide-based vaccines and prior to 2014 these trials contributed to 11.6 % of all trials (Fig. 2a insert). The 2nd most commonly targeted indication, prostate cancer, has been predominantly investigated with dendritic-cell based vaccines (17 out of 46 total registered trials) (Fig. 2 insert 1).

When comparing the top indications examined in trials conducted between 1999–2013 and 2014 onwards we observed a reduction in the proportion of vaccine trials for Melanoma, and an increase in trials targeting Prostate cancer, NSCLC, brain cancers, Pancreatic and Ovarian cancer (Fig. 2b).

The selection of melanoma as primary target for therapeutic vaccines is most likely due to the large number of known melanoma antigens and the natural antigenicity of melanomas (reviewed in [13]). It has been known for a long time that certain tumor types, among which melanoma, are inherently more responsive to immune therapy (i.e., “immunogenic”) than others. For instance, immunotherapy is most often effective against melanomas (reviewed in [1]) which may regress spontaneously [18] or in response to therapy [7] concomitant with autoimmune symptoms. Moreover, current FDA approved “active” immunotherapies for solid tumors [6] are limited to only a few tumor types (e.g., melanoma, bladder, kidney, etc.). Nevertheless, in spite of the large number of trials into melanoma, there remains no FDA approved immunotherapeutic vaccines for the treatment of melanoma [19].

### Registration of trials over time

**Fig. 3:**
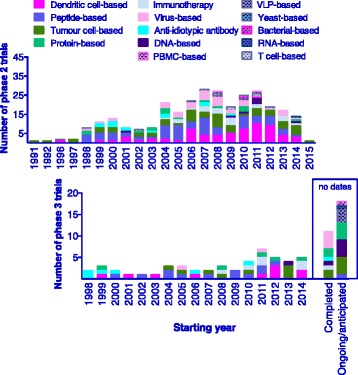
Evolution of Phase 2 and Phase 3 trial registration over time. Using a total of 347 studies where trial start date was available the number of phase 2 and phase 3 trial registered per year since 1999 is shown. Twenty nine phase 3 entries did not include start dates and are sorted based on continuing (ongoing or anticipated) or completed status

The data set for phase 2 and 3 therapeutic vaccine trials revealed a reduction in the number trials after 2011 (Fig. 3), though numbers obtained at the time of analysis for 2015 are most likely underestimated. A cross-sectional analysis of 955 cancer vaccination phase 1–3 trials by Lu et al. [13] revealed a earlier decline, with a decrease in the total number of oncology vaccine trials registered from 2008. This prior analysis also included Phase 1 trials; hence the pattern for an earlier decline is likely due to a decline in phase 1 studies. Interestingly, when we specifically examined the 50 phase 3 trials for which a start date was provided there was an increase in the number of trials registered after 2010 and a steady number of trials registered between 2010–2014. Twenty nine studies did not report start dates however we were able to segregate these as either completed (which includes early termination or suspension) or as ongoing or anticipated (approved but not initiated) based on information from company websites or published information (Fig. 3). These undated values are likely to skew the results but this data suggests that although the number of phase 2 studies may not be increasing, and even decreasing, there is continued investment and efforts in immunotherapeutic vaccines at the phase 3 stage (Fig. 3).

A thorough review of success rates for investigational drugs has been recently published [9] and reported that the percentage of oncology vaccines progressing from phase 2 to 3 stage (39.5 %) was on par with that of oncology product in general (28.3 %), yet still indicating a drop off for vaccine progression between phase 2 to phase 3. However, in the transition between phase 3 to new drug application (NDA) or biologic license application (BLA) although 45.2 % of all oncology products obtained approved, only 8.3 % or (12 entities) of preventative and therapeutic vaccines sought the following milestone towards NDA/BLA (versus 64 % of non-oncology vaccines), confirming that the bottle neck for oncology vaccine progression is late in development rather than early [3].

### Combination treatments in therapeutic vaccine trials

**Fig. 4:**
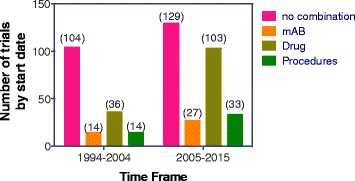
Number of trials including combinations with drugs, mAB or procedures (surgery, radiotherapy, hormone treatment, cell transplants or standard/supportive care) between the decades of 1994–2004 and 2005–2015 for 413 trials where start dates were available

The analysis of therapeutic trials under investigation indicated that combination therapy is common and of the 451 trials reviewed, 185 included a combination with a drug (chemotherapeutic agent), mAB or procedure (surgery, radiotherapy, hormone treatment, cell transplants or standard/supportive care). Over the last two decades, the number of vaccine-only studies were similar, however the last decade has seen an increase in the number of trials with a combination with a mAB, drug or procedure (Fig. 4 and Table 3).

**Table 3:** Combination antibody therapies under investigation

Start year for vaccine trials including mAB combinations																			
	Start year	1997	1998	1999	2000	2001	2002	2004	2005	2006	2008	2009	2010	2011	2012	2013	2014	2015	Total
Anti-idiotypic antibody	3H1					1													1
	Lung					1													1
	BEC2 mab		1	1															2
	Lung		1	1															2
	Racotumomab												1						1
	NSCLC												1						1
Targeted	Bevacizumab											1	2		1	1			5
	Brain															1			1
	Colorectal Cancer												1						1
	Lung												1						1
	Ovarian Cancer														1				1
	Renal Cell Cancer (RCC)											1							1
	Cetuximab								1					1					2
	Colorectal Cancer													1					1
	Pancreatic Cancer								1										1
	Erlotinib													1					1
	NSCLC													1					1
	Trastuzumab (Herceptin)							1	1	1	1	1			1	1			7
	Breast Cancer							1	1	1	1	1			1	1			7
	Rituximab				1			2		1		2							6
	Leukemia									1									1
	Lymphoma				1			2				2							5
Immune-suppressive	Daclizumab													1					1
	Melanoma													1					1
	muromonab-CD3	2						1											3
	Brain	1																	1
	Melanoma	1						1											2
Checkpoint inhibitor	Ipilimumab						1	3		1						1	1		7
	Melanoma						1	3		1							1		6
	Pancreatic Cancer															1			1
	nivolumab																	1	1
	Pancreatic Cancer																	1	1
	Pidilizumab (CT-011)												2	1	1				4
	Leukemia												1						1
	Myeloma												1						1
	Neoplasm														1				1
	Renal Cell Cancer (RCC)													1					1
	Total	2	1	1	1	1	1	7	2	3	1	4	5	4	3	3	1	1	41

145 trials examined vaccination in combination with a chemotherapeutic entity and 47 of these trials specially examined cyclophosphamide treatment. In addition, 19 trials included radiation as a prior/concurrent procedure. Another 41 trials were combined with a monoclonal antibody (mAB) treatment regime, which included treatment with the checkpoint inhibitors anti-PD1 and anti-CTLA-4 as well as tyrosine kinase agonists or anti-VEGF. Details of the mAB investigated are shown in Table 3.

The rationale for the numerous studies investigating the potential synergistic effects of combining vaccines with conventional chemotherapeutic and radiation therapies or with checkpoint inhibitors such as anti-PD1 has been reviewed in [2, 10, 24]. Chemotherapy and radiation are routinely used for the treatment of cancer, and have been shown to promote anti-tumor immunity by inducing immunogenic cell death as part of its intended therapeutic effect and by disrupting strategies that tumors use to evade immune recognition (reviewed [4] and [21]). CPI blockade therapies such as CTLA-4 or PD1/PDL1 inhibitors have recently been approved for melanoma and lung (PD1 inhibitor) cancer treatment and have demonstrated tremendous activity as monotherapies. The co-administration of these monoclonal antibodies and therapeutic vaccines may further increase the number of responders to the therapy, in particular for the patients that have weak natural immune responses to the tumor, or for all tumors that are weakly immunogenic. The therapeutic potential of such combination is currently evaluated in phase 2 and 3 with various vaccine modalities (see Table 4). Though no trend was observed in the number of phase 2 and 3 studies combining CPI blockade and vaccines, it is expected that this number will increase rapidly given the accumulating clinical evidence pointing toward a promising role for checkpoint blocking antibodies in a rapidly expanding spectrum of additional solid tumors including renal cell cancer, ovarian cancer, bladder cancer, head and neck cancer, and gastric cancer.

**Table 4:** Vaccine trials including checkpoint inhibitor mABs

NCT Number	Title	Indication	Sponsor/Collaborators	Phases	Vaccine Type	Vaccine Name	MAB
NCT00077532 (completed)	Monoclonal Antibody With or Without gp100 Peptides Plus Montanide ISA-51 in Treating Patients With Stage IV Melanoma	Melanoma	National Institutes of Health Clinical Center (CC) National Cancer Institute (NCI)	Phase 2	Peptide-based vaccine (adjuvanted)	gp100 antigen in incomplete Freund’s adjuvant	Ipilimumab
NCT00094653 (completed)	MDX-010 Antibody, MDX-1379 Melanoma Vaccine, or MDX-010/MDX-1379 Combination Treatment for Patients With Unresectable or Metastatic Melanoma	Melanoma	Bristol-Myers Squibb	Phase 3	Peptide-based vaccine (multi-peptide)	MDX-1379 (gp100) Melanoma Peptide Vaccine	Ipilimumab
NCT00084656 (completed)	Monoclonal Antibody Therapy and Vaccine Therapy in Treating Patients With Resected Stage III or Stage IV Melanoma	Melanoma	Bristol-Myers Squibb National Cancer Institute (NCI)	Phase 2	Peptide-based vaccine (multi-peptide, adjuvanted)	Tyrosinase/gp100/MART-1 Peptide vaccine in Montanide	Ipilimumab
NCT01067287 (ongoing)	Blockade of PD-1 in Conjunction With the Dendritic Cell/Myeloma Vaccines Following Stem Cell Transplantation	Myeloma	Beth Israel Deaconess Medical Center Dana-Farber Cancer Institute Brigham and Women's Hospital Rambam Health Care Campus Gateway for Cancer Research Department of Defense	Phase 2	Dendritic cell-based vaccine (autologous tumour-fused)	Dendritic Cell/Myeloma Fusion Cell Vaccine with or without CT-011	Pidilizumab (CT-011)
NCT02054520 (ongoing)	Immunotherapy Study for Patients With Stage IV Melanoma	Melanoma	NewLink Genetics Corporation	Phase 2	Tumour cell-based vaccine (allogenic, aGal transfected)	Hyperacute Melanoma Cancer vaccine Dorgenmeltucel-L	Ipilimumab
NCT01096602 (ongoing)	Blockade of PD-1 in Conjunction With the Dendritic Cell/AML Vaccine Following Chemotherapy Induced Remission	Leukemia	Beth Israel Deaconess Medical Center National Institutes of Health (NIH) CureTech Ltd Dana-Farber Cancer Institute	Phase 2	Dendritic cell-based vaccine (autologous tumour-fused)	Dendritic Cell/AML Fusion Cell Vaccine with or without CT-011	Pidilizumab (CT-011)
NCT01441765 (ongoing)	PD-1 Alone or With Dendritic Cell/Renal Cell Carcinoma Fusion Cell Vaccine	Renal Cell Cancer (RCC)	Beth Israel Deaconess Medical Center National Institutes of Health (NIH) National Cancer Institute (NCI) Dana-Farber Cancer Institute	Phase 2	Dendritic cell-based vaccine (autologous tumour-fused)	Dendritic Cell/Renal Cell Carcinoma Fusion Cell Vaccine with or without CT-011	Pidilizumab (CT-011)
NCT01420965 (ongoing)	Sipuleucel-T, CT-011, and Cyclophosphamide for Advanced Prostate Cancer	Neoplasm	Georgia Regents University	Phase 2	Dendritic cell-based vaccine (autologous tumour-pulsed)	Provenge (Sipuleucel-T/APC8015) + Drug: CT-011 (Anti-PD1 Antibody)	Pidilizumab (CT-011)
NCT01896869 (ongoing)	A Phase 2, Multicenter Study of FOLFIRINOX Followed by Ipilimumab With Allogenic GM-CSF Transfected Pancreatic Tumor Vaccine in the Treatment of Metastatic Pancreatic Cancer	Pancreatic Cancer	Sidney Kimmel Comprehensive Cancer Center	Phase 2	Tumour cell-based vaccine (allogenic, GM-CSF transfected)	GVAX	Ipilimumab
NCT02243371 (ongoing)	GVAX Pancreas Vaccine (With CY) and CRS-207 With or Without Nivolumab	Pancreatic Cancer	Sidney Kimmel Comprehensive Cancer Center Bristol-Myers Squibb Stand Up To Cancer Aduro BioTech AACR Research Acceleration Network	Phase 2	Tumour cell-based vaccine (allogenic, GM-CSF transfected), Bacteria-based vaccine (Listeria)	GVAX	nivolumab
NCT00032045 (completed)	Vaccine Therapy and Monoclonal Antibody Therapy in Treating Patients With Stage IV Melanoma	Melanoma	National Cancer Institute (NCI)	Phase 2	Peptide-based vaccine (adjuvanted)	gp100 antigen incomplete Freund’s adjuvant	Ipilimumab
*NCT00357461 (withdrawn prior to enrolment)*	*Ipilimumab With or Without Vaccine Therapy in Treating Patients With Previously Treated Stage IV Melanoma*	*Melanoma*	*Bristol-Myers Squibb National Cancer Institute (NCI)*	*Phase 2*	*Peptide-based vaccine (multi-peptide, adjuvanted)*	*gp100:209-217(210M) & gp100:280-288(288V) peptide vaccine in incomplete Freund’s adjuvant*	*Ipilimumab*

If proven successful in clinical trials, these combination therapies could offer a multi-pronged attack against cancers not adequately treated with a single treatment modality.

### Redefining success in immunotherapeutic vaccine trials

In the studies examined, the vast majority of Phase 2 and Phase 2 trial protocols included Efficacy or Safety and Efficacy as an endpoint (Additional file 1: Table S2) however an important question and issue that remains to be solved is whether conventional endpoints such as clinical response, time to progression, disease/progression free progression and overall survival are suitable or ideal for assessing the efficacy of immunotherapies. Is the expectation that an oncology vaccine should be measured with the same endpoints as conventional treatments appropriate? Provenge, the only FDA approved therapeutic vaccine achieved a 4.1-month improvement in median survival (25.8 months in the sipuleucel-T group vs. 21.7 months in the placebo group) but did not show evidence for an improvement in time to disease progression [13]. A review by [22] on the FDA approval of anti-neoplastic agents between 2002–2012 indicated that the majority (64 %) of regular approvals did not rely on overall survival as the end point, but rather on progression free survival (PFS), time to progression (TTP) or response rates. It is now being acknowledged that the measure of immunotherapeutic activity may not fit the linear activity timeline for more ‘immediate’ acting conventional chemotherapeutic treatments (discussed in [11, 12]). The response to immunotherapeutic activity may be delayed, and preceded by transient increases in tumor burden due to TILs (generally deemed to mark progressive disease and drug failure) prior to detection of a clinical response. In addition, immunotherapies may exert effects against new lesions rather than primary lesions. The recognition that immunotherapies often result in a delayed separation of survival curves also means that alternative statistical analysis that detects events from the point of separation should be applied to account for this delay [12]. These observations have been considered in the revised immune-related response criteria that accounts for baseline and new lesions and changes in total tumor burden over different time points [25]. In addition, identifying appropriate the endpoints to determine successful outcomes in cancer immunotherapy trials, may be an important step in progressing this well-studied but up till now, seemingly unfruitful field.

## Conclusion

From this review it is apparent that over the last 15 years, there has been substantial efforts in developing therapeutic vaccines encompassing various platforms. Although only one vaccine, Provenge, has achieved FDA approval, there is nevertheless continued activity in the development of therapeutic vaccines, including Phase 3 trials. Despite the low approval rates for therapeutic vaccines, an appreciation of the minimal toxicity associated with immunotherapy compared to conventional treatments and the continued need to find treatments for cancers not adequately treated with current therapies provide the motivation to stay in the course to pursue development of more effective immunotherapeutic vaccines and combination treatment regimes. Past learnings from previous trials which have lead to the adoption of improved endpoint criteria better suited to determining a clinical response to immunotherapy and improved statistical measurements capable of detecting an outcome to immunotherapy feed the hope that ongoing efforts will eventually yield improved trial outcomes and ultimately bring benefit to the patients. In addition, the recent approval and therapeutic success of CPI blockade therapies have opened a new era for immunotherapies, which may, when paired with vaccines, improve their efficacy and consequently likelihood of approval.

## Additional file

Additional file 1: Table S1. Vaccine categories in trial completed prior to 2013, or with planned completion post 2013. **Table S2.** Endpoints included in Phase 2 & Phase 3 trial protocols. **Table S3.** Platform, adjuvant and antigen-loading variants within vaccine categories. (DOCX 39 kb)

## Abbreviations

Ab: Antibody
CC: Colon cancer
GBM: Glioblastoma Multiforme
GM-CSF: Granulocyte-macrophage colony-simulating factor
OS: Overall survival
PFS: Progression free survival
RFS: Recurrence free survival
RRC: Renal cell carcinoma
TTP: Time to progression
mAB: Monoclonal Antibody
TIL: Tumor infiltrating lymphocytes
