# Supplementary material for: A quantitative analysis of therapeutic cancer vaccines in phase 2 or phase 3 trial
Source: J Immunother Cancer. 2015 Nov 17;3:48. doi: 10.1186/s40425-015-0093-x (PMC4647658; doi:10.1186/s40425-015-0093-x)
Supplement: Additional file 1: Table S1. — Vaccine categories in trial completed prior to 2013, or with planned completion post 2013. Table S2. Endpoints included in Phase 2 & Phase 3 trial protocols. Table S3. Platform, adjuvant and antigen-loading variants within vaccine categories. (DOCX 39 kb) [file 40425_2015_93_MOESM1_ESM.docx]

| **Primary completion dates (year) based on vaccine category** | | | | | |
| --- | --- | --- | --- | --- | --- |
| **Vaccine category (completion year)** | **Phase 2** | **Phase 2/3** | **Phase 3** | **Grand Total** | **Ratio of Phase 3:Phase 2** (Number of phase 3 trials/phase 2 trials) |
| **Anti-idiotypic antibody vaccine** | **13** | **1** | **5** | **19** | **1:38** |
| 2000-2013 | 13 | 1 | 4 | 18 | 1:31 |
| 2015 | 0 | 0 | 1 | 1 | *NA* |
| **Bacterial-based vaccine** | **2** |  |  | **2** | ***NA*** |
| 2013 | 1 |  |  | 1 | *NA* |
| 2018 | 1 |  |  | 1 | *NA* |
| **Dendritic cell-based vaccine** | **64** | **3** | **7** | **74** | **1:11** |
| 2003-2013 | 30 | 0 | 3 | 33 | 1:10 |
| 2014-2024 | 34 | 3 | 4 | 41 | 1:12 |
| **DNA-based vaccine** | **7** |  | **1** | **8** | **1:14** |
| 2004-2013 | 2 | 0 | 0 | 2 | *NA* |
| 2014- 2017 | 5 | 0 | 1 | 6 | 1:20 |
| **PBMC-based vaccine** | **2** |  |  | **2** | *NA* |
| 2011 | 1 |  |  | 1 | *NA* |
| 2016 | 1 |  |  | 1 | *NA* |
| **Peptide-based vaccine** | **69** | **1** | **11** | **81** | **1:16** |
| 1999-2013 | 49 | 1 | 6 | 56 | 1:12 |
| 2014-2020 | 20 | 0 | 5 | 25 | 1:25 |
| **Protein-based vaccine** | **15** |  | **5** | **20** | **1:33** |
| 2000-2013 | 12 | 0 | 2 | 14 | 1:17 |
| 2014-2018 | 3 | 0 | 3 | 6 | 1:100 |
| **RNA-based vaccine** | **1** |  |  | **1** | *NA* |
| 2015 | 1 |  |  | 1 | *NA* |
| **T cell-based vaccine** | **11** |  |  | **11** | *NA* |
| 2004-2013 | 7 | 0 | 0 | 7 | *NA* |
| 2015-2018 | 4 | 0 | 0 | 4 | *NA* |
| **Tumor cell-based vaccine** | **56** | **2** | **6** | **64** | **1:11** |
| 2001-2013 | 34 | 0 | 3 | 37 | 1:9 |
| 2014-2023 | 21 | 2 | 4 | 27 | 1:19 |
| **Virus-based vaccine** | 33 | 1 | 1 | 35 | **1:3** |
| 2002-2013 | 23 | 1 | 0 | 24 | *NA* |
| 2014-2019 | 10 | 0 | 1 | 11 | 1:10 |
| **VLP-based vaccine** | 1 |  |  | 1 | *NA* |
| 2009 | 1 |  |  | 1 | *NA* |
| **Yeast-based vaccine** | 2 |  |  | 2 | *NA* |
| 2014 | 2 |  |  | 2 | *NA* |
| **Grand Total** | **276** | **8** | **36** | **320** | **1:13** |

Additional file 1

Table S1 Vaccine categories in trial completed prior to 2013, or with planned completion post 2013.

Table S**2 Endpoints included in Phase 2 & Phase 3 trial protocols**

|  | **Trial Endpoint** | | | |  | |
| --- | --- | --- | --- | --- | --- | --- |
|  | **Safety** | **Efficacy/Safety & Efficacy** | **Not provided** | **Number of trials/phase** | |  |
| Phase 2 | 0.30% | 94% | 6% | 364 | |  |
| Phase 2 Phase 3 |  | 92% | 8% | 12 | |  |
| Phase 3 |  | 71% | 29% | 72 | |  |

Table S3 Platform, adjuvant and antigen-loading variants within vaccine categories

| **Variants of Peptide-based, Dendritic-cell based, Tumor-cell based vaccines and virus/bacteria/yeast based categories** | | | | | |
| --- | --- | --- | --- | --- | --- |
| **Vaccine category** | **Variant details** | **Phase 2** | **Phase 2 Phase 3** | **Phase 3** | **Grand Total** |
| **Peptide-based vaccine** | multi-peptide, adjuvanted | 40 | 1 | 5 | 46 |
|  | adjuvanted | 36 |  | 8 | 44 |
|  | liposome | 7 |  | 3 | 10 |
|  | no details | 4 |  | 1 | 5 |
|  | multi-peptide | 3 |  | 1 | 4 |
|  | adjuvanted, peptide-pulsed | 1 |  |  | 1 |
|  | adjuvanted, Protein | 1 |  |  | 1 |
|  | peptide, + DNA-based plasmid | 1 |  |  | 1 |
| **Dendritic cell-based vaccine** | autologous tumor-pulsed | 30 |  | 6 | 36 |
|  | peptide-pulsed | 13 |  | 1 | 14 |
|  | allogenic tumor-pulsed | 8 |  | 1 | 9 |
|  | autologous tumor-fused | 4 |  |  | 4 |
|  | RNA-pulsed | 4 |  |  | 4 |
|  | autologous tumor-pulsed, Tumor stem cell vaccine allogenic |  | 3 |  | 3 |
|  | protein-pulsed | 3 |  |  | 3 |
|  | autologous RNA |  |  | 2 | 2 |
|  | virus transduced | 2 |  |  | 2 |
|  | no details | 1 |  |  | 1 |
|  | allogenic idiotype-pulsed | 1 |  |  | 1 |
|  | autologous tumor SC-pulsed | 1 |  |  | 1 |
|  | autologous tumor-fused, peptide-pulsed | 1 |  |  | 1 |
|  | autologous tumor-fused/tumor-phagocytosed | 1 |  |  | 1 |
|  | autologous tumor-pulsed, Anti-idiotypic antibody vaccine | 1 |  |  | 1 |
|  | autologous tumor-pulsed, DNA-based vaccine | 1 |  |  | 1 |
|  | idiotype-pulsed | 1 |  |  | 1 |
|  | peptide-pulsed, Immunotherapy T cells | 1 |  |  | 1 |
|  | viral vector transfected, Immunotherapy SCT | 1 |  |  | 1 |
| **Tumor cell-based vaccine** | allogenic, GM-CSF transfected | 15 |  | 2 | 17 |
|  | autologous | 14 |  | 3 | 17 |
|  | allogenic | 7 | 1 | 3 | 11 |
|  | allogenic, aGal transfected | 7 | 1 | 2 | 10 |
|  | autologous, GM-CSF transfected | 6 |  |  | 6 |
|  | allogenic, GM-CSF transfected, Bacteria-based vaccine Listeria | 3 |  |  | 3 |
|  | allogenic, TGFb transfected | 1 |  | 1 | 2 |
|  | autologous tumor, T cell-based vaccine autologous, Immunotherapy anti-CD3 | 2 |  |  | 2 |
|  | allogenic, HLA-A2/4-1BB transfected |  | 1 |  | 1 |
|  | allogenic, Dendritic cell-based | 1 |  |  | 1 |
|  | allogenic, Immunotherapy aMILs, | 1 |  |  | 1 |
|  | allogenic, autologous | 1 |  |  | 1 |
|  | autologous tumor lysate | 1 |  |  | 1 |
|  | autologous, liposome | 1 |  |  | 1 |
|  | autologous, Immunotherapy activated T cells | 1 |  |  | 1 |
|  | autologous, Immunotherapy dendritic cells | 1 |  |  | 1 |
|  | B7-1 modified | 1 |  |  | 1 |
|  | IR prostate cancer cells | 1 |  |  | 1 |
| **Virus-based vaccine** | Vaccinia & Fowlpox | 20 |  | 4 | 24 |
|  | MVA | 10 | 1 | 1 | 12 |
|  | ALVAC | 6 |  |  | 6 |
|  | adenovirus | 4 |  |  | 4 |
|  | ALVAC, Peptide-based vaccine adjuvanted | 1 |  |  | 1 |
|  | CMV | 1 |  |  | 1 |
|  | Fowlpox | 1 |  |  | 1 |
|  | Fowlpox, T cell-based vaccine autologous | 1 |  |  | 1 |
|  | oncolytic | 1 |  |  | 1 |
|  | Vaccinia | 1 |  |  | 1 |
| **Protein-based vaccine** | adjuvanted | 17 | 1 | 12 | 30 |
|  | no details | 2 |  |  | 2 |
| **T cell-based vaccine** | autologous, tumor-pulsed | 3 |  | 1 | 4 |
|  | TCR, Dendritic cell-based vaccine peptide-pulsed | 3 |  |  | 3 |
|  | allogenic,autologous tumor-pulsed | 1 |  |  | 1 |
|  | anti-idiotypic antibody | 1 |  |  | 1 |
|  | autologous, peptide-pulsed | 1 |  |  | 1 |
|  | autologous, peptide-pulsed, Peptide-based vaccine adjuvanted | 1 |  |  | 1 |
|  | peptide-pulsed, Dendritic-cell based vaccine peptide-pulsed | 1 |  |  | 1 |
|  | transgenic | 1 |  |  | 1 |
| **Bacterial-based vaccine** | Listeria | 2 |  | 1 | 3 |
| **PBMC-based vaccine** | autologous peptide-pulsed | 1 |  |  | 1 |
|  | autologous protein-pulsed | 1 |  |  | 1 |
| **Yeast-based vaccine** | S. cerevisiae | 2 |  |  | 2 |
